# Diffusion-Controlled Nucleation, Growth, and Self-Assembly of Silica Nanoparticles in Laminar Microfluidic Flow

**DOI:** 10.1021/acs.langmuir.5c05489

**Published:** 2026-04-10

**Authors:** Nícolas M.C. Gomes, Fernanda T.J. Serrão, Matheus B. Wilges, Arquiminio B.S. Neto, Gustavo H.S. Domingos, Marco A.L. Cordeiro

**Affiliations:** † Graduate Program in Materials Science and Engineering, Federal University of São Carlos, Rod. Washington Luis, São Carlos 13565-905, Brazil; ‡ Department of Materials Engineering, Federal University of São Carlos, Rod. Washington Luiz, São Carlos, São Paulo 13565-905, Brazil

## Abstract

Microfluidic devices have emerged as a promising platform for controlling small fluid volumes at the micrometer scale, offering new opportunities in several areas, especially in the synthesis of nanomaterials such as SiO_2_ nanoparticles. This potential is related to the precise control of synthesis parameters such as temperature, reagent concentration, flow rate, and micromixing, which are difficult to control in batch synthesis. Furthermore, the spatial confinement regime can facilitate the separation of nucleation and growth steps, providing superior control over particle size and dispersion. However, their operability under challenging conditions, such as high reagent concentrations, and the mechanisms governing synthesis, growth, and self-assembly remain insufficiently understood. Therefore, this study compares microfluidic and batch syntheses of SiO_2_ nanoparticles. Through numerical simulations of laminar flow and species advection diffusion, we map the precursor overlap along the microchannel, including the identification of a narrow interdiffusion region where nucleation is expected to initiate. The role of flow in the formation of new self-assembled structures is also discussed, as consistent with the size-dependent hydrodynamic characteristics of particles under laminar convection and enhanced by confinement.

## Graphical abstract

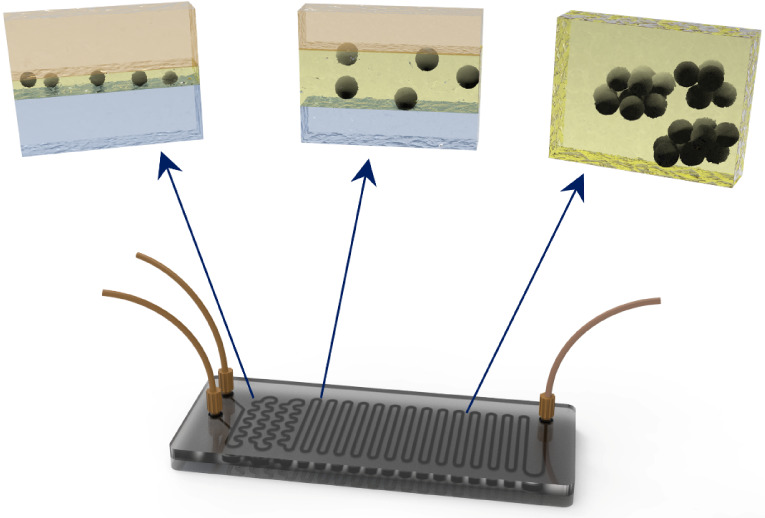

## 1 Introduction

Microfluidic devices have emerged as a promising tool for controlling small volumes of fluids at the micrometer scale, enabling important advances in diagnostics, drug discovery and delivery, cell-based assays, organs-on-chips, and materials synthesis.^1–6^ Among these opportunities, microfluidic devices have the potential to improve or even enable new nanoparticle (NP) synthesis protocols by precisely controlling key synthesis parameters such as temperature, reagent concentration, flow rate, and component mixing.^5–8^ This versatility of these devices can be further enhanced by the possibility of designing them with different mixing approaches, with passive or active micromixing structures, or operating as laminar coflow without dedicated mixers.^9^ For example, although both batch and microfluidic routes can achieve adequate final solution mixing, they do so through distinct mechanisms, promoting distinct NP synthetic outcomes. While the batch route relies on stirring-induced convective mixing to homogenize the reactor volume, the microfluidic route can enable precise control over reactant transport in confined microscale flows, either using micromixing structures or operating as laminar coflow with a confined interdiffusion layer, providing greater control over reactant transport and concentration gradients in the reaction media.^10–12^ In particular, microfluidic devices operated as laminar coflow with a confined interdiffusion layer can provide a transport-based handle on the burst nucleation and subsequent growth by limiting transverse mixing to molecular diffusion across the stream interface.^8,10,13^ These regimes are conveniently described by the Reynolds (*Re*) and Péclet (*Pe*) numbers, which are the ratio of inertial to viscous forces in a flow and the ratio of advective to diffusive transport for a species.^14^ However, on one hand the confinement regime promotes better optimization of reactant mixing in the transverse direction in the channels, on the other hand the longitudinal dynamics of reactant diffusion and solution flow can make NP nucleation and growth more complex.^8,15^ It is generally stated that this dynamic can still be hampered by the use of higher reagent concentrations, which can promote uncontrolled nucleation, growth and agglomeration of particles. If aggregates are formed, they can ultimately obstruct the channels and interrupt further synthesis, leading to failure of the microfluidic system.^8,15,16^ Consequently, understanding these parameters is fundamental for developing new NPs synthesis protocols.

Silica (SiO_2_) is one of the NPs that could benefit from new synthesis protocols using microfluidic devices. Due to the technological importance of these particles (e.g., drug delivery, bioimaging, environmental remediation, catalysis, biosensing, protective and antifouling coatings),^17–21^ combined with their tunable physical and chemical properties and ease of surface functionalization, microfluidic devices have the potential to offer improved control over the synthetic parameters for the production of SiO_2_ NPs, allowing further enhancement of their properties. The most common synthetic route in batch approaches for the production of SiO_2_ NPs is the so-called Stöber method.^17,18,22^ This approach is based on sol–gel synthesis, in which a silica precursor, typically tetraethyl orthosilicate (TEOS, Si­(OC_2_H_5_)_4_), is hydrolyzed in an alcoholic solution (usually methanol or ethanol), mediated by the catalytic action of ammonia. Depending on the synthesis parameters (e.g., concentration and ratio of reactants and catalyst, temperature, aging time), it is possible to obtain particles ranging from tens of nanometers to microns in diameter, with a variable size distribution depending on those conditions.^17,18,23–26^ On the other hand, due to the complexity of the interactions among reactants and the reaction parameters, it is common to find disagreement on how these parameters are related to the final product. For example, although it is known that increasing the concentration of TEOS and ammonia in the reaction medium leads to an increase in the average SiO_2_ particle size, under certain conditions, these trends can change. Owing to the interplay among the reactants, TEOS and ammonia can have antagonistic effects, and modify the final average particle size and its size distribution.^23,24,27,28^ Even the agitation of the reaction medium is another factor that can influence the size and size dispersion in batch processes, although rarely highlighted in studies.^17,29^ Although batch reactions require continuous stirring to homogenize the medium throughout the process, stirring also increases collision frequency among particles and thus promotes growth by coalescence.^17^

Microfluidic devices have gained great attention in the synthesis of SiO2 and other metal oxides since they can overcome several challenges associated with traditional batch synthesis such as broader particle size distribution, less control over the nucleation process and higher reagent consumption.^6^ Differently from batch processes, the confined and laminar transport in microfluidic routes stabilize the interfacial reaction zones, which reduces uncontrolled micromixing and modulates the complexity of interactions between reactants. This improves the reaction reproducibility, particularly at high precursor concentrations. Nevertheless, despite the possibilities offered by microfluidic devices, there are still uncertainties about their effective superiority over batch synthesis under high precursor content regimes, particularly in terms of particle size dispersion and propensity to channel clogging.^30^ Accordingly, the present study comparatively investigates the synthesis of SiO_2_ nanoparticles using batch and microfluidic approaches, with emphasis on reaction dynamics and reagent concentration regimes typically considered challenging for microfluidic systems.

## 2 Experimental Section

### 2.1 Materials

Tetraethyl orthosilicate (TEOS, Si­(OC_2_H_5_)_4_) (98%; Aldrich), absolute ethanol (P.A.; Exodo Cientifica), ammonium hydroxide (NH_4_OH) (30% in water, Synth), isopropyl alcohol (99.5%.; Quemis), light-curing resin (3D Standard, 3DLAB), polydimethylsiloxane (PDMS) (Sylgard 184 Elastomer Kit, Dow). All of the chemicals were used without further purification.

### 2.2 Microfluidic Device Fabrication

Polydimethylsiloxane (PDMS)-based microfluidic devices were fabricated using soft lithography from 3D-printed molds. The microfluidic device was designed using computer-aided design (CAD) software, consisting of two solution inlets, channels with a 700 μm × 700 μm cross-section, and a total length of 700 mm, as shown in Figure S1. Additionally, a support was 3D printed to contain both the resin and the positive mold, and to control the thickness of the final device (Figure S1). Both the support and the positive mold were 3D printed using an LD-002H printer (UV 405 nm; Creality) with a 2K LCD screen (1620 × 2560 pixels), following the printing parameters recommended by the resin manufacturer. After 3D printing, an additional UV curing step was performed using a UW-01 rotating platform (Creality) to ensure complete polymerization of the resin.

For the casting process, PDMS resin was stirred for 5 min, poured into the 3D-printed molding assembly (mold and containment structure), and then placed in a desiccator under vacuum for 15 min to remove air bubbles. The assembly was then placed in an oven at 50 °C for 16 h to cure the PDMS resin. Finally, the cured PDMS was demolded and placed onto a thin, flat, surface-treated glass substrate treated with plasma. An additional heat treatment at 100 °C in an oven for 30 min was carried out to ensure complete adhesion of the cured PDMS to the glass substrate. Figure 1 shows the step-by-step manufacturing of the microfluidics device.

**Figure 1:**
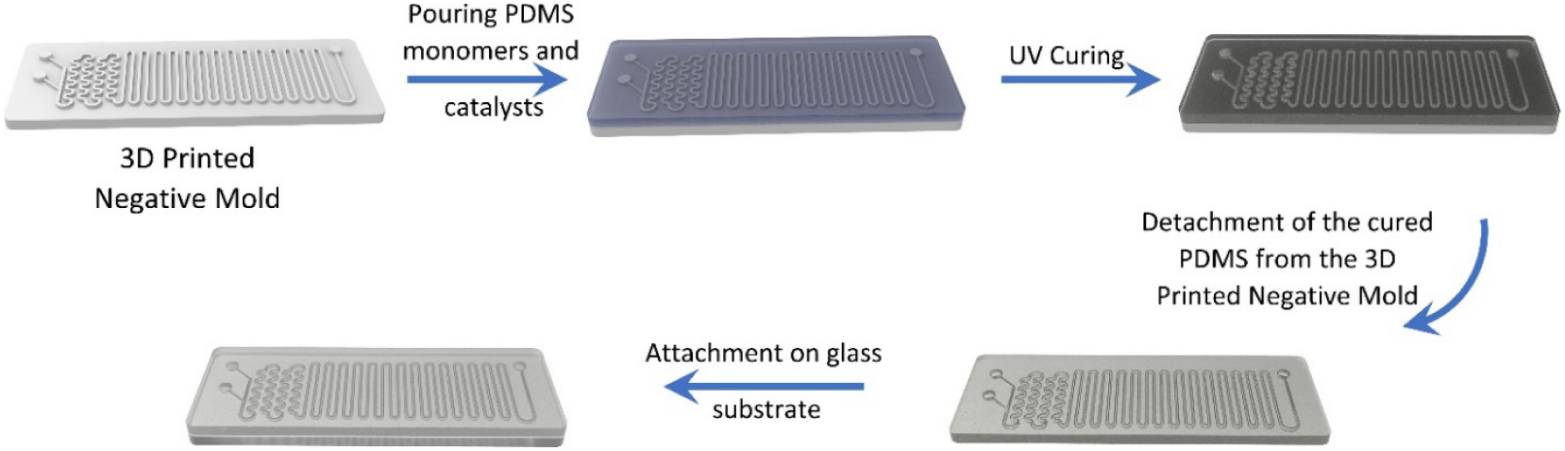
Step-by-step process for manufacturing the microfluidic devices, from material preparation through final assembly.

### 2.3 SiO_2_ Synthesis Protocols

The synthesis of silica NPs was carried out according to the Stöber method,^22^ which involves the hydrolysis of tetraethyl orthosilicate (TEOS) in the presence of water and a low-molecular-weight alcohol, catalyzed by ammonia, followed by subsequent condensation. Specifically, this study focused on reagent concentrations that are typically considered problematic in microfluidic synthetic routes due to channel fouling, particularly TEOS and ammonia, as these reagents exert the greatest influence on the final SiO_2_ particle size for the parameters used in this study.^17,31^ Water concentration is another important factor in SiO_2_ synthesis. However, studies have shown that variations in water concentration at higher ranges (>5 mol L^–1^) become less relevant to the reaction, resulting in negligible variations in particle size and dispersion.^31^ Therefore, since the water concentration in this study was consistently above 9 mol L^–1^, water had little influence under these conditions, and the study focused on TEOS and ammonia.

For the syntheses, two ethanolic stock solutions were prepared: one containing TEOS (1.3 mol L^–1^) and the other containing ammonia (1.3 mol L^–1^) and water (17.2 mol L^–1^). According to the desired concentrations for each reaction run, these solutions were diluted with ethanol, resulting in concentrations according to Table 1. Since this study focused on the influence of ammonia and TEOS, the relative proportions of ammonia and water were kept constant during reactions and diluted with ethanol accordingly. In order to compare the approaches, the syntheses were performed by conventional sol–gel synthesis (CS) and microfluidic synthesis (MS) methods to produce SiO_2_ particles. For MS approach, two syringes controlled the flow of the solutions at the inlets of the microfluidic device, with a total inlet flow rate of 15 μL/min (according to the concentrations in Table 1). The fluid residence time from the inlet to the outlet of the microfluidic device was approximately 20 min. After leaving the microfluidic system, the mixture remained in a reservoir for an additional 40 min, in order to accumulate around 0.9 mL of the mixture. All MS were carried out at 25 °C. Additionally, for sample MA, the same concentration as sample M2 was used, but a total flow rate of 40 μL/min. Although it has a shorter residence time inside the microreactor (∼7.5 min), it was collected after 52.5 min in the reservoir so that it could be compared with sample M2 in relation to the same experiment time. Furthermore, despite the high concentration conditions, the clogging phenomenon did not occur in a way that hindered the experiments while the microreactor was operational. Using the same concentrations and reaction time, the synthesis of SiO_2_ particles was performed using the CS approach. For this synthesis, the TEOS solution was added dropwise to the ammonia solution under vigorous magnetic stirring in a beaker, with subsequent reaction at 25 °C for 1 h (the same time required for MS). For both protocols, immediately after synthesis, the produced particles were separated by centrifugation (14000 rpm) and washed three times with ethanol to remove possible unreacted precursors.

**Table 1:** Identification of Samples According to TEOS and Ammonia Concentrations for CS and MS Syntheses

	Concentration (mol L^–1^)		
Samples	TEOS	NH_4_OH	H_2_O
C1	0.7	1.3	17.2
C2	1.0	1.0	13,2
C3	1.3	0.7	9.3
M1	0.7	1.3	17.2
M2	1.0	1.0	13.2
M3	1.3	0.7	9.3

### 2.4 Characterization

The microstructure of the SiO_2_ particles was characterized by scanning electron microscopy (SEM) using a Magellan 400L (FEI, operating at 5 kV with a working distance of 4 mm). Samples were prepared by drop-casting 25 μL of each suspension onto an aluminum substrate, allowed to dry naturally, and then sputter-coated with a thin layer of gold to reduce charging effects. The average particle size of each synthesized sample was calculated by measuring at least 300 particles with ImageJ from SEM micrographs acquired at different regions of each sample. The frequency and size distribution of each sample were plotted as violin plots. Further statistical analyses of the particle size data were performed with Gaussian mixture modeling (GMM) to determine the optimal number of modes via the Bayesian information criterion (BIC), and by computing Ashman’s *D* and the overlap coefficient to quantify mode separation and distribution overlap. The mean ± SD values presented reflect the overall dispersion of the particle size distribution as measured by SEM images, and should not be interpreted as experimental variability between trials, which is assessed separately by the inter/intraexperimental variance ratio presented above. For multimodal samples, GMM component means and SDs are reported to describe within-component dispersion and should not be interpreted as measurement uncertainty of SEM. Further details on the methodology and results are presented in the Supporting Information. In order to ensure reproducibility, for each condition described in Table 1, the procedure was performed at least three times in a completely independent manner using freshly prepared solutions. Especially for the MS samples, each microreactor was used only once to avoid any carryover effects. The ratio between inter- and intraexperimental variances confirmed the reproducibility of the process, since in all cases $\frac{\sigma_{INTER}}{\sigma_{INTRA}}\ll 1$ . Fourier transform infrared spectroscopy (FTIR) analysis of the SiO_2_ particles was performed using a Shimadzu IRPrestige-21 spectrometer equipped with an attenuated total reflectance (ATR) accessory. Prior to FTIR measurements, the samples were kept overnight in a desiccator.

### 2.5 Transport Simulations

Numerical simulations of fluid dynamics and mass transport in microfluidic devices were performed using COMSOL Multiphysics software. For the simulations, two-dimensional (2D) cross sections of the device channels were modeled based on the actual device dimensions, using the same layouts used in the production process. These simulations address only hydrodynamics and species transport; they do not include reaction kinetics, nucleation, growth, or particle transport. Because the fluids in the channels of the studied processes have a low Reynolds number (*Re* < 1), turbulent flow is not expected, and transverse mixing occurs mainly by molecular diffusion. Thus, the laminar behavior of the fluids and the diffusion of the reactants involved in the process were addressed by adding the “Laminar Flow” and “Transport of Dilute Species” interfaces to the software’s simulation protocol, to solve the Navier–Stokes and convection-diffusion equations, respectively. Furthermore, the fluids were defined as incompressible Newtonian fluid with no-slip boundary conditions on the channel walls. Although the diffusing species have about the same magnitude of diffusion coefficient (∼10^–9^ m^2^/s, 25 °C), ammonia was selected as the representative species in the simulation because it exhibits the largest diffusion coefficient among reactants in ethanol and water mixtures (1.5 × 10^–9^ m^2^/s, 25 °C), providing an upper limit for transverse interdiffusion without altering qualitative conclusions.^32,33^

## 3 Results and Discussion

Figure 2 shows a representative microfluidic device fabricated for this study. The device showed high optical transparency and well-defined microchannels, which allowed visual inspection of the channel network and verification of device integrity before running the synthesis experiments.

**Figure 2:**
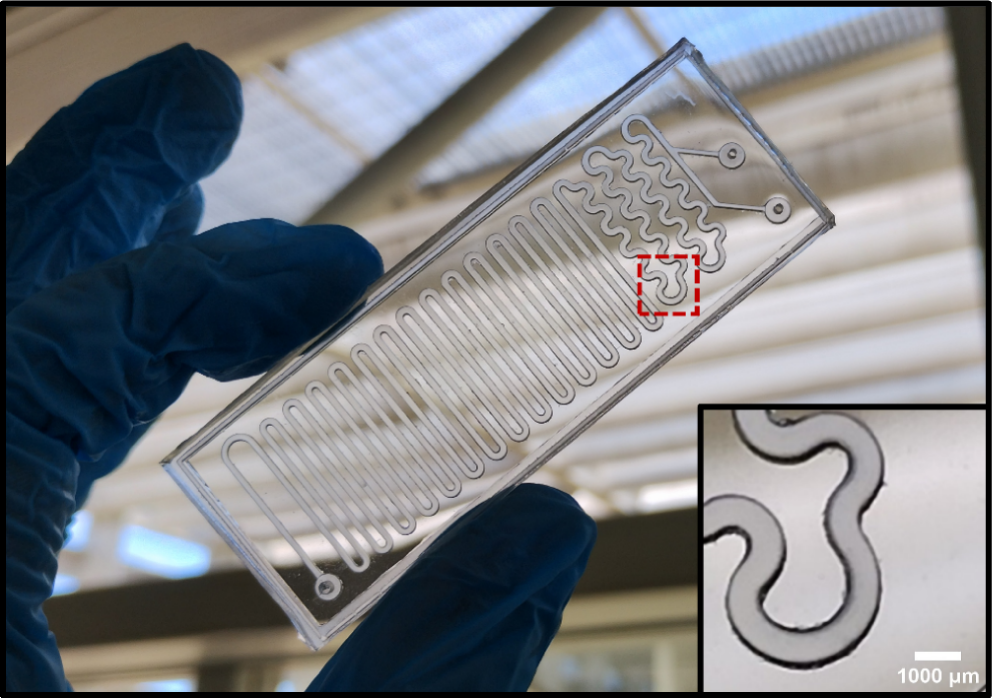
Transparent microfluidic device produced. The inset shows a magnified view of a representative region of the microchannel network (dashed region), highlighting the channel features.

Figure 3 shows the SEM images of the SiO_2_ nanostructures synthesized by the microfluidic method (MS) and by conventional synthesis (CS), according to the precursor concentrations listed in Table 1. An overall shift in central tendency is observed in the CS samples as TEOS concentration increases: 141 ± 35 nm, 320 ± 206 nm, and 418 ± 193 nm for C1, C2, and C3, respectively.

**Figure 3:**
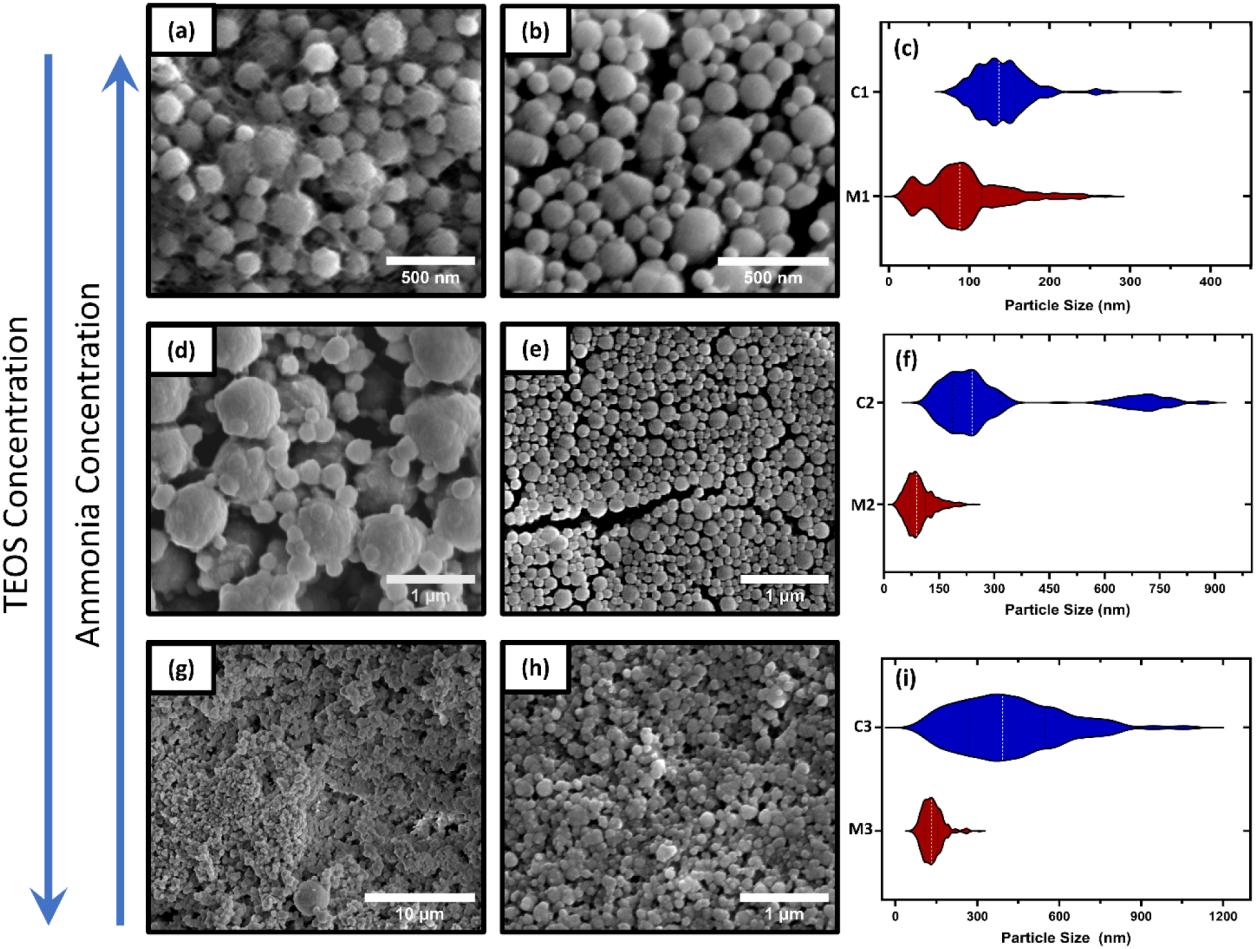
SEM images of the SiO_2_ particles produced by CS and MS routes, according TEOS and ammonia concentrations, and their respective particle size distribution. (a) C1 sample; (b) M1 sample; (c) Particle size distribution of the C1 and M1 samples. (d) C2 sample; (e) M2 sample; (f) Particle size distribution of the C2 and M2 samples; (g) C3 sample; (h) M3 sample; (i) Particle size distribution of the C3 and M3 samples.

Although other studies have reported an increase in average particle size with increasing TEOS concentration,^31^ the distributions observed here are broad and substantially overlapping. Therefore, the discussion focuses on shifts in central tendency rather than on clearly separated size populations. This outcome is consistent with the experimental design of this study, which intentionally explored higher precursor concentrations than those commonly used to obtain narrower Stöber size distributions. Classical Stöber syntheses can produce SiO_2_ particles with great size control and often narrow distributions. However, the resulting size distribution strongly depends on the reaction conditions. In fact, recent studies demonstrate that the effects of precursor effects (TEOS, NH_3_, and H_2_O), solvent characteristics, and synthesis temperature are neither independent nor linear, since the interrelationships among the reagents can modify or even reverse the particle size trends and their distributions.^17,18,23,31^ In general, reaction regimes with lower precursor concentrations (typically below 0.5 mol L^–1^) often produce narrower distributions, while broader, bimodal, or even aggregated populations can arise from more concentrated systems.^28,31^ For example, Wang et al.^31^ reported concentration-dependent size distribution regimes and distinct aggregation behaviors according to the initial TEOS concentration. TEOS concentrations lower than 0.56 mol L^–1^ resulted in bimodal particle size distributions, while monodisperse particles were obtained for concentrations between 0.67 and 1.115 mol L^–1^, and aggregation occurred above 1.24 mol L^–1^. Similarly, Fernandes et al.^17^ obtained particles ranging from ∼9 to ∼800 nm, using a multivariate factorial design encompassing TEOS concentrations from 0.31 to 0.54 mol L^–1^ and NH_3_ from 0.18 to 0.35 mol L^–1^, demonstrating that even moderate increases in precursor concentrations can generate substantial size variability. Thus, the wide distributions observed in this study are consistent with operation in an even higher concentration regime.

While earlier studies suggested that the influence of TEOS concentration is almost negligible in the lower range (0.02–0.5 M),^17,18,25^ more recent findings suggest that higher TEOS concentrations promote larger particles size due to the greater availability of monomers in the reaction.^17,24,34^ In the present study, which explored an even higher concentration range (0.7–1.3 M), TEOS also increased the average size of the particles in the CS synthesis method, but with distinct behavior and magnitude. In comparison, sample C1 produced smaller particles due to the limited amount of monomer in the growth phase, which was mostly consumed during the initial hydrolysis stage (i.e., nucleation). As the TEOS concentration increased, the availability of monomers for further particle growth also increased, resulting in larger particle sizes for C2 and C3 (details of hydrolysis and condensation mechanisms are provided in Figure S2).

Ammonia interacts synergistically with the TEOS, acting as a catalyst in both hydrolysis and condensation reactions. Ammonia increases the hydrolysis rate by providing hydroxide ions in the ethanol–water media (from the NH_3_/NH_4_^+^ equilibrium) solution, a stronger nucleophile than water.^18,35^ These radicals promote the nucleophilic attack at silicon and cleavage of Si–OEt bonds, leading to the formation of silanol groups (Si–OH). Subsequently, ammonia, facilitates the deprotonation of silanols (Si–OH ⇌ Si–O^–^ + H^+^) to generate siloxide ions (Si–O^–^), which accelerates siloxane condensation (Si–O^–^ + Si–OR/Si–OH → Si–O–Si), enhancing the condensation process and accelerating particle growth.^18,25,35^ Therefore, variations in the TEOS/ammonia ratio directly affect the availability of monomers and the rate of nucleation versus growth, which ultimately control the final particle size and distribution. FTIR spectra collected for all samples confirmed the characteristic SiO_2_ vibrational bands (Si–O–Si stretching and bending modes), with only minor variations in O–H and H–O–H bands related to surface silanols and adsorbed water (Figure S3).

Thus, lower TEOS concentration and higher ammonia concentration (C1) will favor faster hydrolysis over condensation, resulting in smaller particles, as there are no monomers left for the growth phase. Higher concentrations of TEOS and lower concentrations of ammonia (C3) will result in larger particles because monomers are available during the whole process (i.e., nucleation and growth), which led to a broader particle size distribution. Besides, an interesting result occurred in the condition with intermediate concentrations of TEOS and ammonia (C2) (Figure 3d). In this case, most of the monomers were consumed in the hydrolysis, leaving fewer monomers for the condensation phase. However, the ammonia concentration was high enough to activate the particle surface (by generating siloxane ions) and the later growth process occurred by coalescence of the particles (see Supporting Information Section 5), evidenced by the bimodal distribution of particles. In fact, the particle size distribution of the C2 sample (Figure 3f) shows two main modes: one between 100 and 370 nm (mode mean of 217 nm, with a within-mode SD of 52 nm), and other between 670 and 900 nm (mode mean of 714 nm, with a within-mode SD of 57 nm). This second peak can be attributed to a series of particle coalescences, which interpretation is consistent with the statistical modeling, where GMM–BIC selected *k* = 2 for C2 and Ashman’s *D* ≥ 2 with low overlap indicating well-separated modes (Table S01), supporting a coalescence-driven second population. On the other hand, although ammonia can activate particle surfaces at high concentrations, at even higher concentrations it can enhance deprotonation of silanol groups (Si–OH → Si–O^–^), creating repulsion between particles and consequently decreasing aggregation growth, as observed in sample C1 (Figure 3a,c).^35,36^ This phenomenon can be further intensified by the formation of an electrical double layer on the particle surface, with the first layer consisting of NH_4_^+^ ions attracted to the negatively charged silica surface and the second layer composed of negatively charged ions, such as OH^–^. When two particles in this system approach each other, the double layers overlap and electrostatic repulsion increases, hindering the coalescence process.^37^

Fernandes et al.^17^ demonstrated that, individually, ammonia and TEOS have the most significant effects on the size of SiO_2_ particles synthesized by the Stöber method, while water has a moderate influence and ethanol has a low influence. Nevertheless, this synergy between ammonia and water does not significantly influence the syntheses because the ammonia-to-water molar ratio was kept constant throughout the syntheses, with dilution achieved by the addition of ethanol.^17^ This behavior is consistent with prior studies with higher water concentrations (6–10 mol L^–1^), in which variations in this concentration range exerts a negligible influence on the average size of the SiO_2_ particles synthesized by the Stöber method.^23,31^

In contrast to CS routes, the SiO_2_ particles synthesized by the MS route proceeded in a quite different manner. The TEOS and ammonia concentrations had a milder influence on the average particle sizes and their dispersions in the MS samples: 94 ± 49 nm, 100 ± 39 nm, and 134 ± 36 nm for M1, M2, and M3, respectively. Despite the progressive increase in the average particle size with increasing TEOS concentration, this effect was considerably less pronounced than for the particles synthesized by the CS route. The ammonia concentration also had a mild influence on the MS route, even at higher concentrations, with a slight increase in the coefficient of variation of mean particle sizes with increasing concentration. The particle size distribution of sample M1 can be described as partially overlapping subpopulations: one between 15 and 50 nm (mode mean of 32 nm, with a within-mode SD of 16 nm) and another between 50 and 120 nm (mode mean of 84 nm, with a within-mode SD of 9 nm), with an additional smaller fraction at ∼140 nm. Sample M1 (Figure 3b,c) exhibited a multimodal and partially overlapping distribution, with GMM–BIC favored *k* = 3. When forcing *k* = 2, the Ashman value indicates possible bimodality (*D* ≈ 1.9) and high modal overlap (∼0.57), suggesting two main modes, and a third mode related to a tail of larger particles (Table S1). It is consistent with the formation of nuclei in a confined region, with subsequent coalescence processes by activation of the particle surfaces by the increased ammonia concentration (similar to the behavior of sample C2), and a residual growth by additional monomers. Although growth processes by monomer addition and coalescence may occur in samples M2 and M3, there is a prevalence of a single dominant mode with narrower dispersion, and a weak tail not supported by model selection, indicating a more uniform growth process. In comparison, in CS syntheses, increasing TEOS and decreasing NH_4_OH resulted in larger particles, while the opposite trend resulted in smaller particles. In contrast, in MS syntheses, variations in the precursors produced only small variations in the average particle size, largely within the experimental uncertainty (Table S01, Figures S5 and S6). Despite these differences between the synthetic routes, the FTIR spectra of the samples indicate the same SiO_2_ chemical signature for samples from both methods (Figure S3).

One of the most important characteristics of the microfluidic system is its flow behavior, which governs reactant transport and the conditions associated with particle formation. In order to understand this process, it was performed flow simulations to analyze reactant concentration flows during the SiO_2_ synthesis under MS conditions. For the simulations, the ammonia concentration profile is displayed throughout the process as a representative model of the diffusing species. This choice is justified by the fact that all reactants have mass diffusion coefficients of the same order of magnitude (∼10^–9^ m^2^/s), with ammonia at the upper end; the use of ammonia therefore sets an upper limit for interdiffusion without altering the qualitative trends.^32,33^ Figure 4 shows the steady state ammonia concentration field in the microfluidic device for the MS conditions. The key outcome is the predicted thickness and downstream evolution of the interdiffusion layer, which defines the region of reactant coexistence during the early stages of synthesis. The first characteristic observed is the fluid flow pattern and its influence on the concentration profile during the early stages of synthesis (highlighted in each simulation in Figure 4). Throughout the microchannels, the flow remained mainly laminar, with the fluid moving in parallel layers with minimal mixing among them initially; inertial forces are negligible compared to viscous forces, leading to a predictable flow pattern. This regime is the most commonly found in microfluidic processes where the Reynolds number is small (*Re* < 1), which also characterizes this study. In this range, flow characteristics are weakly dependent on the tortuosity of simpler channels, even in geometries such as the serpentine design used here.^38–40^ Numerical investigations indicate that significant turbulent regions only occur in systems with much higher Reynolds numbers or with more complex microchannels geometries.^10,39,41^ Furthermore, the Péclet number (*Pe*), which quantifies the ratio between convective and diffusive transport of solutes, is well above unity in the systems studied here (*Pe* ≫ 1), indicating that convective transport dominates the diffusion of entities $(Pe_{NH_{3}}\approx 238$ , *Pe*_TEOS_ ≈ 714). This combination of low Reynolds number and high Péclet numbers not only points to the formation of laminar flows, but also the predominance of axial advection over all the transverse diffusion of precursors.

**Figure 4:**
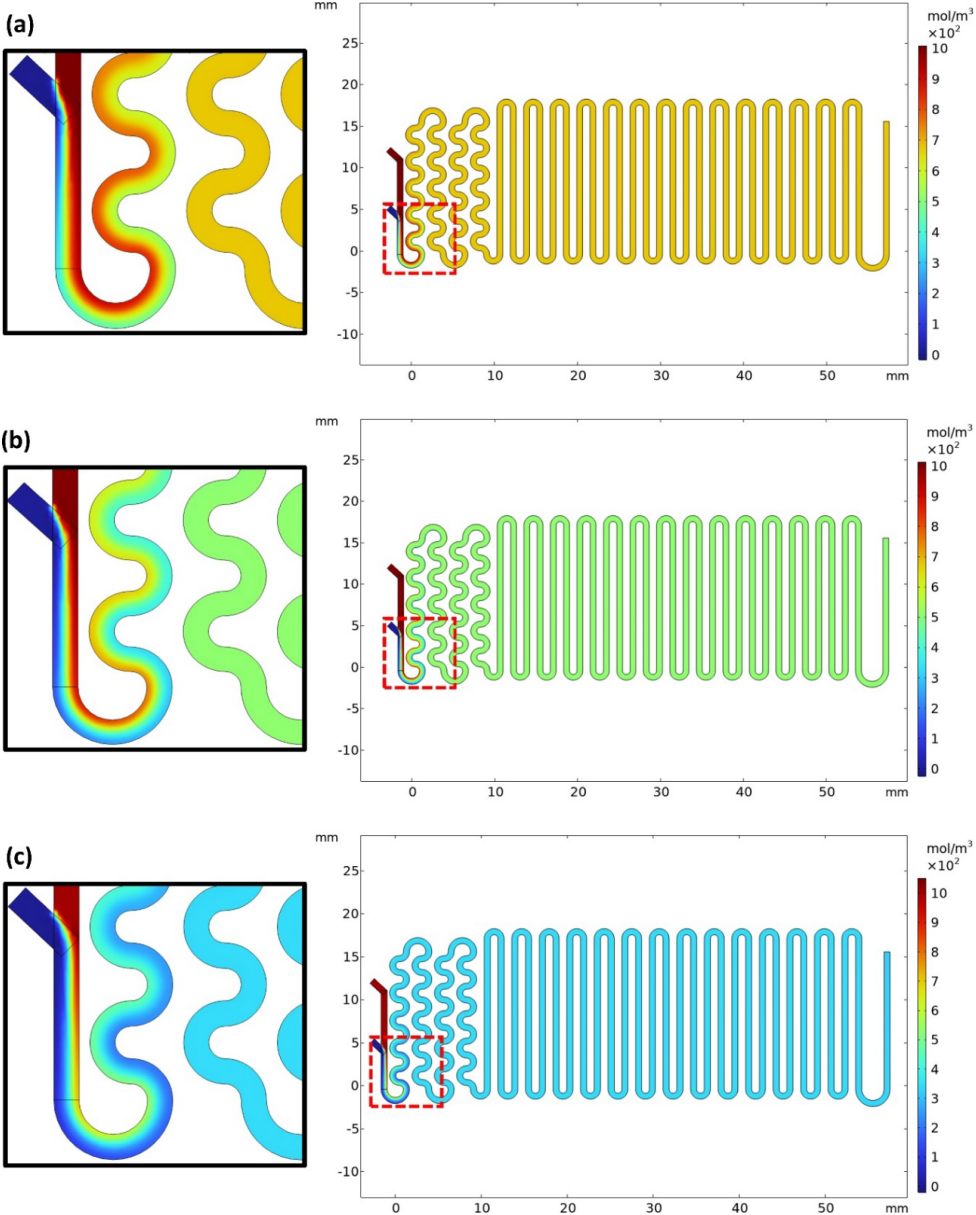
Simulation of the ammonia concentration profile in the microfluidic synthesis devices after flow process stabilization for (a) M1, (b) M2, and (c) M3 synthesis conditions. Each image includes a highlighted rectangular region, which is magnified on the left of the image for better visualization of the simulation.

Although the numerical simulations address only fluid dynamics and species transport, and not the reaction process itself (i.e., reaction kinetics, nucleation, and growth are not directly modeled), it is possible to infer, from the coupling between the simulated interdiffusion pattern of precursors and the particle size distributions observed by SEM, the spatial onset and extent of the nucleation and growth processes of SiO_2_ particles. The narrow interdiffusion zone between the two precursor solutions defines the region where the local coexistence of sufficient reactant concentrations can first generate the supersaturation required for initial nuclei formation, consistent with classical nucleation theory. With the widening of this interdiffusion region along the microchannels, there is a progressive availability of precursors for subsequent particle growth. From classical nucleation theory, the broader size distributions in CS samples could arise from heterogeneous nucleation, which occurs in regions with a lower Gibbs free energy barrier (e.g., vessel walls, impurities, and within macroscopic concentration gradients).^42–44^ Reactions via the MS route confine reactant overlap to the diffusive mixing layer (Figure 5a), which can reduce spatial variations in supersaturation and lower the probability of uncontrolled nucleation events, even at high TEOS concentrations. As the synthesis continues, the growth process proceeds as interdiffusion of precursor solutions increases (Figure 5b). The growth process of the SiO_2_ particles remains confined to a restricted interfacial region, which prevents random monomer addition to particle surfaces and results in smaller particles with a narrower size distribution. In the final step of the process (aging, Figure 5c), particle size growth due to monomer addition is reduced, and controlled coalescence may occur, resulting from the activation of particle surfaces by a higher ammonia concentration (as seen for sample M1, Figure 3c).

**Figure 5:**
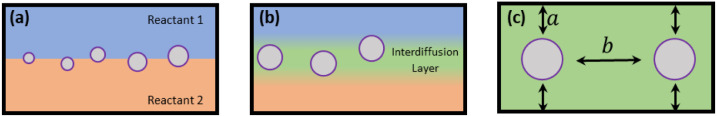
Scheme of the particle synthesis by MS route. (a) Initial stage; (b) intermediate stage and (c) final stage.

All previous syntheses of the MS route were performed using a total inlet flow rate of 15 μL/min of reagent solutions (combined flow rate of both inlet streams), varying only the reagent ratio. However, the flow rate in the microfluidic system can also affect the synthesis process. In order to investigate the influence of this parameter, a new SiO_2_ synthesis was performed with a 1:1 TEOS to ammonia ratio (identical to sample M2) but with a total flow rate of 40 μL/min, hereafter denoted MA. The SEM images of this sample (Figure 6a,b) show not only a change in particle size but also the formation of a distinct particle arrangement. Compared to M2, the mean particle diameter increased significantly to 245 ± 40 nm, while the narrow particle size distribution and lower coefficient of variation (Figure 6c) suggest that nucleation and growth remained under controlled conditions. Owing to understanding this behavior, flow simulations were also performed to analyze the ammonia concentration profile for MA (Figure S4). Compared to M2, the simulations reveal that a steeper concentration gradient persists over a greater longitudinal distance along the microchannels, increasing the amount of monomers during the growth phase, possibly resulting in greater particle growth. On the other hand, the nucleation and growth phase remain confined to a narrow reactant interdiffusion zone, since *Re* stays below unity and *Pe* increases, reinforcing the laminar regime with limited transverse mixing.

**Figure 6:**
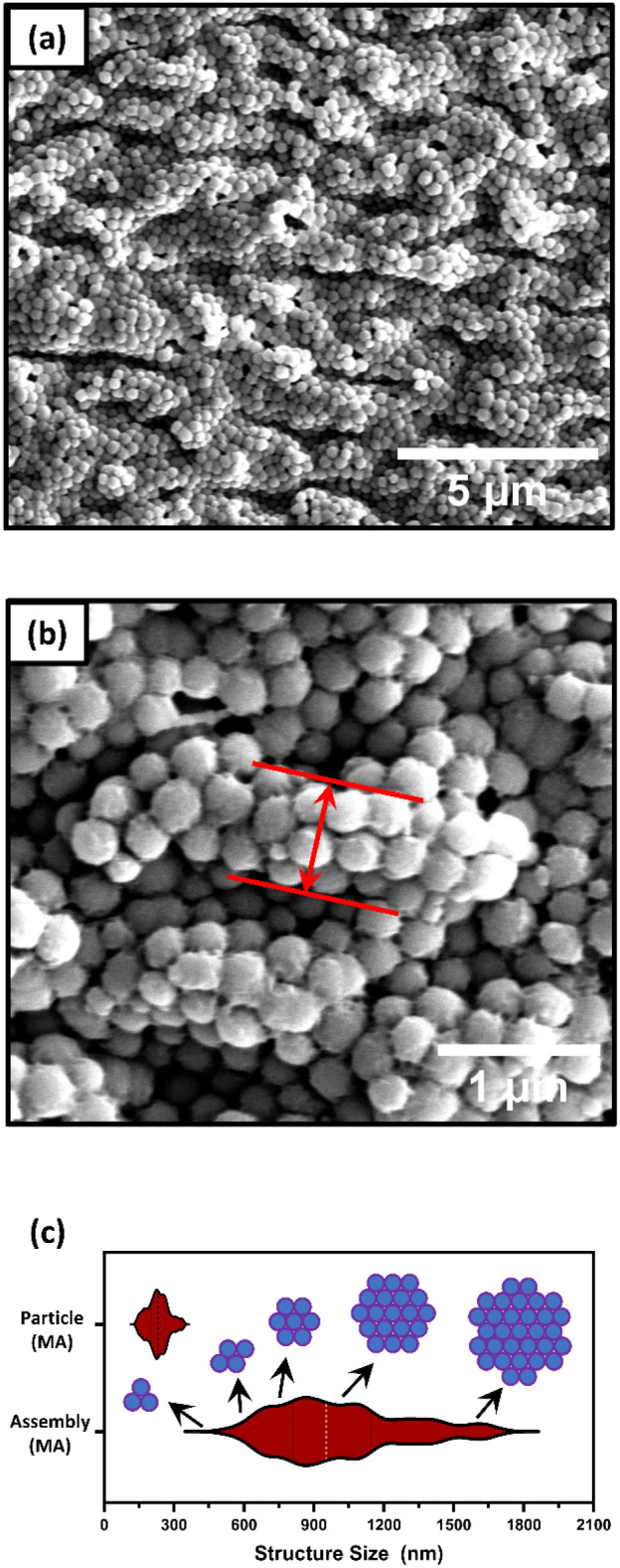
(a) and (b) SEM images of the SiO_2_ particles of the MA; (c) particle size distribution and particle assembly size distribution.

To highlight the differences in reagent concentrations (in this case, ammonia) along the microchannels, a pixel-by-pixel concentration difference map (Δ*C*(*x*,*y*)) was computed between the two numerical simulations (MA and M02), according to

1 $$\Delta C\left(x,y\right)=C_{MA}\left(x,y\right)-C_{M2}\left(x,y\right)$$

where *C*_MA_(*x*,*y*) and *C*_M2_(*x*,*y*) denote the local reagent concentrations at the spatial coordinates (*x,y*) within the channel cross-section for samples MA and M2, respectively. The resulting map (Figure S3c,d) initially shows almost no difference in concentration between the simulations. However, after a short distance along the microchannels, two regions with distinct concentrations appear between the simulations: one with a higher concentration (reddish) and the other with a lower concentration (bluish), separated by a narrow band where the signal difference is zero (black region). This indicates that, although the higher flow rate maintained higher ammonia concentrations over a longer path, the width of the interdiffusion region between the two cases remained essentially unchanged. That is, the flow rate did not significantly alter the thickness of the interdiffusion layer, providing the same spatiotemporal control over the nucleation and growth processes in both syntheses, regardless of the flow rate. Moreover, although the Reynolds number increased slightly (but still below 1, *Re* ≈ 0.67), the Peclet number became even larger, ( $Pe_{NH_{3}}\approx 635$ , *Pe*_TEOS_ ≈ 1905) increasing the tendency for convective laminar transport of reactants along the longitudinal direction over the transverse diffusion.

A second characteristic of sample MA is the formation of ordered particle assemblies in grape-like arrangements. These structures exhibit a close-packed pattern, with no clear preference for face-centered cubic (FCC) or hexagonal close-packing (HCP). Interestingly, the size distribution of these structures (based on the widths highlighted in Figure 6b and quantified in Figure 6c) allows us to infer the number of particles contributing to the packing dimension. The assemblies were found to consist predominantly of 3 to 31 particles, with the majority comprising clusters of 7 to 19 particles.

Self-assembly of particles is one of the most promising bottom-up engineering strategies for creating functional materials, bridging the nano/microlevel interactions and macroscopic material properties. In general, these organizations can be promoted by environmental changes (e.g., temperature variation, solvent evaporation, pH, and ionic strength) and/or by programmable interactions at the nanoscale (e.g., specific interactions like molecular recognition, hydrogen bonding), which can direct the formation of highly ordered architectures.^45,46^ Microfluidic-based colloidal self-assembly also relies on these mechanisms; but, while additional features such as shear gradients, spatial confinement, and temporal control introduce additional complexity in the system, those additional features offer unprecedented opportunities for tuning and directing the assembly process.^47–49^

In this study, a distinct self-assembly mechanism is observed. During the early stages of MA synthesis, the nuclei have small diameters and are reasonably dispersed throughout the system, following laminar streamlines with negligible resistance to motion. However, as the particles grow, particle hydrodynamics becomes significant in laminar advection, intensified by the increased fluid flow in the microfluidic system during MA synthesis. According to Stokes’ Law, larger particles experience stronger drag forces (i.e., a particle’s response to flow is inversely proportional to its radius) and reduced diffusion. Indeed, the Einstein-Stokes relationship indicates that the diffusion coefficient decreases as particle radius increases (i.e., larger particles become progressively more constrained within the flow).^50–52^ Additionally, the progressive increase in particle packing promotes an effective hydrodynamic sieving effect: large and tightly packed particles create additional resistance in the flow, acting as mobile barriers that locally reduce the flow, favoring particle proximity and accumulation.^53^ This accumulation can also modify the laminar flow regime, but since nucleation and growth events due to monomer addition are reduced in the last part of the microfluidic path, there are no detrimental effects on particle size distribution. On the other hand, this local hydrodynamic perturbation can promote better particle packing.

Consequently, the final stage of the MA synthesis can be characterized by the progressive approximation of particles (decrease in distance *b* in Figure 5c), accompanied by a reduced free path for the fluid (decrease in distance *a* in Figure 5c). These phenomena enhance particle packing, and the combined approximation and accommodation processes promote the formation of grape-like structures. Thus, it is inferred that the observed self-assembly is flow-induced and confinement-assisted. Furthermore, additional interactions may contribute to the formation of these structures. For example, if the ammonia concentration is high enough, the particle surfaces can be activated, thus reinforcing the cohesion of the assemblies. Overall, the formation of grape-like ordered assemblies demonstrates a shift from the predominantly Brownian motion common in batch syntheses to flow-directed aggregation, thereby opening opportunities for the engineering of colloidal photonic architectures and other hierarchical materials.

## 4 Conclusions

This study provided a comparative investigation of SiO_2_ NPs synthesis using batch and microfluidic routes, with an emphasis on concentration regimes typically considered challenging for microfluidic systems. First, the influence of reactant concentrations was analyzed, which had a pronounced effect in the CS route. Variations in the TEOS/ammonia ratio directly affected monomer availability and the rates of nucleation and growth throughout the synthesis. Higher TEOS concentrations produced larger particles by sustaining monomer supply during nucleation and growth, which broadened the particle size distribution. Higher ammonia concentrations increased the rate of hydrolysis during the nucleation phase and promoted particle surface activation, thereby intensifying the condensation and accelerating growth. Excess ammonia increased the deprotonation of silanol groups, creating repulsion between particles and, consequently, reducing aggregation-driven growth.

Conversely, variations in reactant concentration had only a mild influence on particle size and dispersion in the MS route, with no signs of clogging or significant particle deposition within the channels. Numerical simulations indicated that the laminar flow regime (*Re* < 1, *Pe* ≫ 1) confined the reaction initially to a narrow interdiffusion region. Although reaction kinetics were not modeled, this confined overlap provides a transport-based explanation consistent with the absence of clogging and with the observed particle size distributions even at high precursor concentrations.

Additionally, numerical simulations demonstrated that higher flow rates in the MS routes did not alter the confinement regime or the initial width of the interdiffusion zone; thus, spatiotemporal control over nucleation and growth steps was maintained. On the other hand, higher flow promoted a late-stage particle organization into grape-like assemblies. This self-assembly was consistent with size-dependent hydrodynamic responses under laminar advection, reinforced by confinement.

Together, the analysis of transport regimes in laminar coflow, alongside the demonstrated self-assembly, highlight the capabilities of microfluidic devices for NP synthesis under high precursor concentrations. The results suggest that microfluidic synthesis is a versatile platform to regulate nucleation, growth, and self-assembly, even at high precursor concentrations, enabling new protocols for other oxide systems and facilitating the integration of continuous-flow synthesis with scalable manufacturing.

## Supplementary Material



## Notes

CAD design of the mold, reaction mechanisms involved in the Stöber method, numerical simulation of the ammonia concentration profile in the microfluidic device for MA sample, and descriptive statistics (PDF)
