# Supplementary material for: Diffusion-Controlled Nucleation, Growth, and Self-Assembly of Silica Nanoparticles in Laminar Microfluidic Flow
Source: Langmuir. 2026 Apr 10;42(21):14611–21. doi: 10.1021/acs.langmuir.5c05489 (PMC13235637; doi:10.1021/acs.langmuir.5c05489)
Supplement: Supplementary file 1 [file la5c05489_si_001.pdf]

Supporting Information

Diffusion-Controlled Nucleation, Growth, and Self-Assembly of Silica Nanoparticles in Laminar Microfluidic Flow

Nícolas M. C. Gomes[1]*; Fernanda T.J. Serrão*[1]*; Matheus B. Wilges*[1]*; Arquiminio B. S. Neto*[1]*;*

Gustavo H. S. Domingos[1]*; Marco A. L. Cordeiro*[2,*]

[1] Graduate Program in Materials Science and Engineering, Federal University of São Carlos, Rod. Washington Luis, São Carlos 13565-905, Brazil.

[2] Department of Materials Engineering, Federal University of São Carlos, Rod. Washington Luiz, São Carlos, São Paulo, 13565-905, Brazil

1. **Microfluidic Device Fabrication**

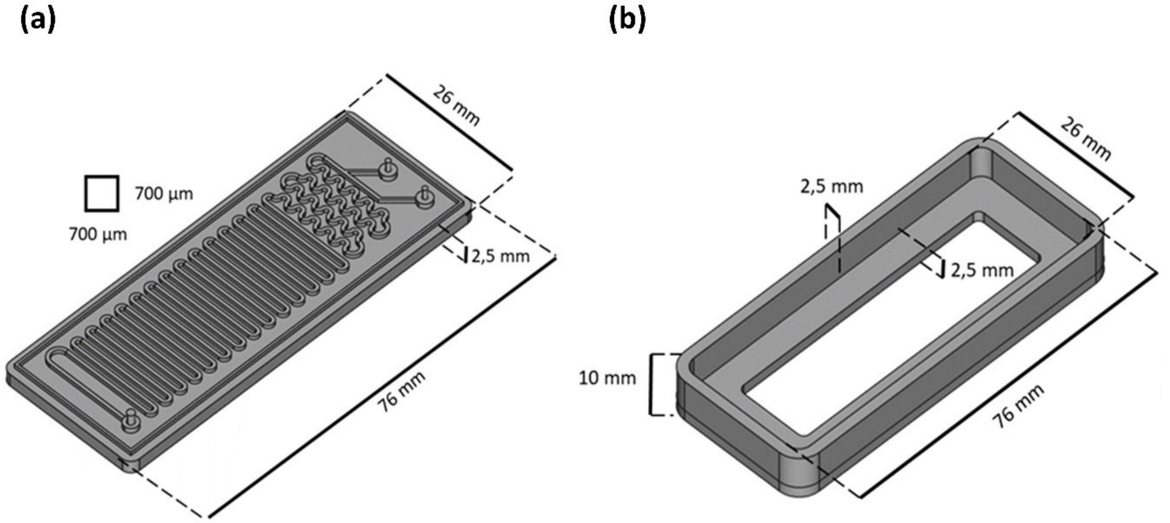

Figure S1 – (a) Illustration of the CAD design of the mold with 700 × 700 µm cross-sectional square channels and (b) of the mold holder to align the mold and control device thickness, with dimensions shown in each panel.

2. Reactions- Stöber Method

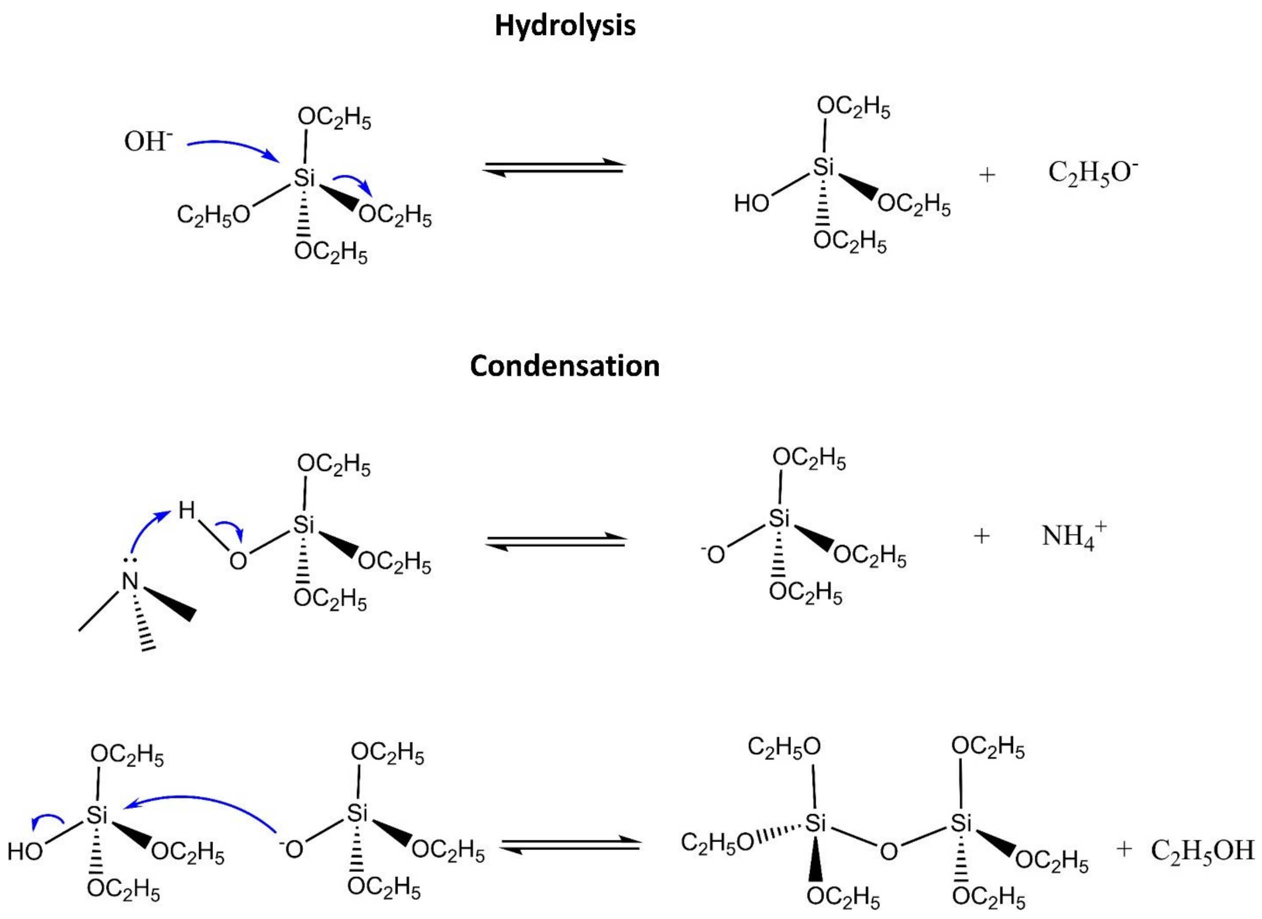

Figure S2- Reaction mechanisms involved in the Stöber method for the synthesis of SiO_2.

3. FTIR spectrum

Figure S3 shows the FTIR spectra of all SiO_2 samples prepared by the batch and microfluidic routes. All spectra display the characteristic silica vibrations, including the intense asymmetric Si–O–Si stretching band at ~1060 cm^{-1}, the symmetric Si–O–Si stretching band at ~790 cm^{-1}, and the Si–O–Si bending mode at ~450 cm^{-1}. A band near ~950–970 cm^{-1} is assigned to surface silanol groups (Si–OH). The broad O–H stretching feature (~3200–3600 cm^{-1}) and the H–O–H bending band (~1640 cm^{-1}) indicate adsorbed water and surface hydroxylation. Minor differences among samples are mainly observed in the relative intensity of the O–H and H–O–H bands, consistent with variations in residual hydration and surface silanol population, while the silica framework bands remain unchanged across all conditions.

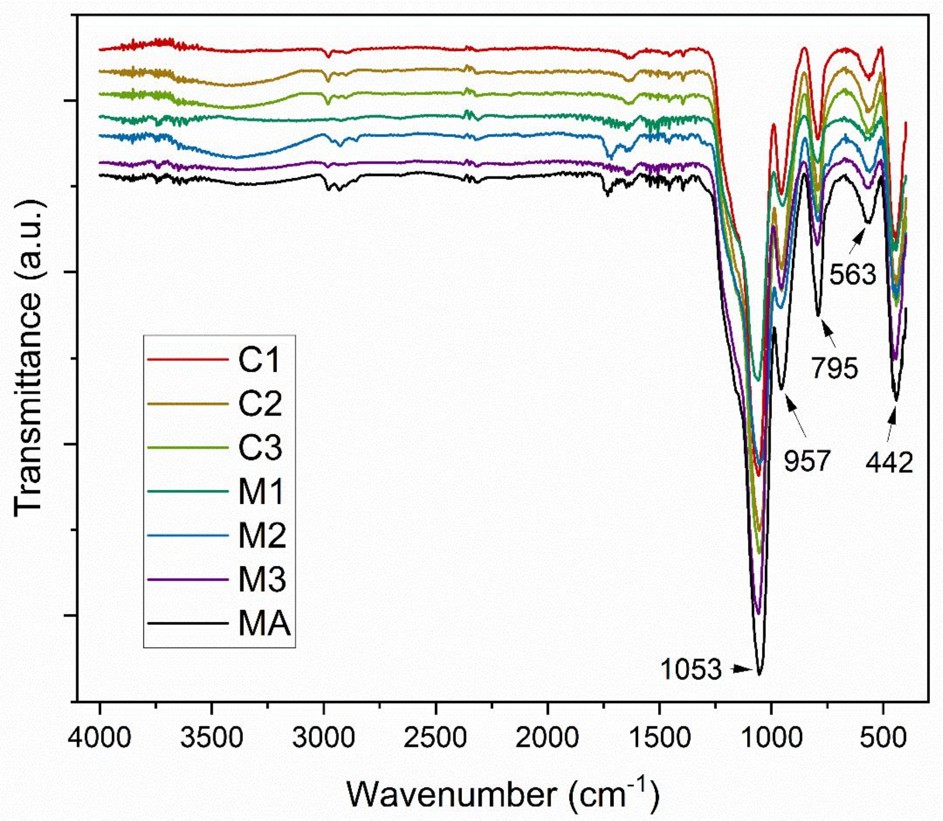

Figure S3: IR spectrum for the different SiO_2 samples collected indicated peaks associated with Si-O-Si bond stretching vibration modes in SiO_4 tetrahedral units at 1053 cm^{-1}, non-bonding Si-OH stretching or stretching due to defects in the structure at 957 cm^{-1}, and Si-O-Si bond stretching in adjacent tetrahedra at 795 cm-1 to 442 cm^{-1}.

4. Numerical Simulation

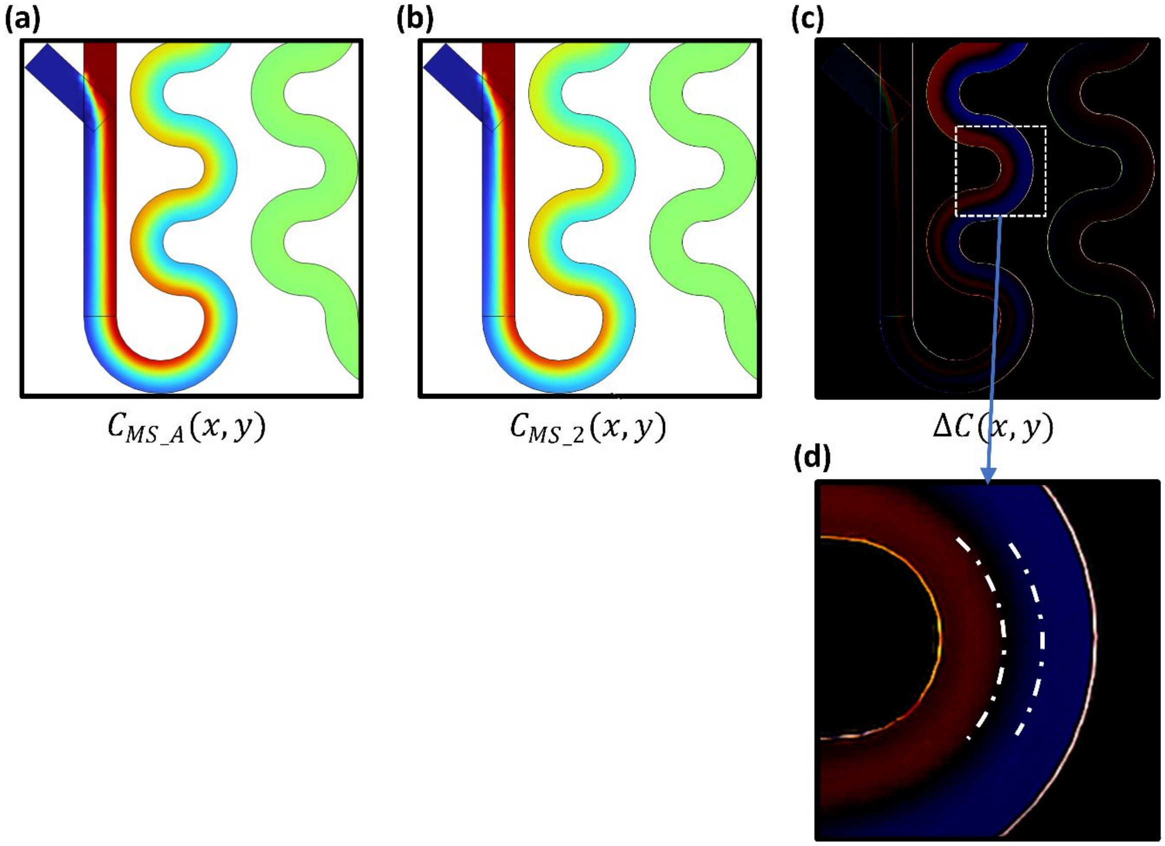

Figure S4- Numerical simulation of the ammonia concentration profile in the microfluidic device for (a) MA and (b) M2 at steady state; (c) pixel-by-pixel concentration difference map of images (a) and (b), where the dark band marks the interdiffusion layer with a difference close to zero; (d) highlight of the highlighted region in (c)

5. Descriptive Statistics

In addition to the mean size, standard deviation (SD), interquartile range (IQR) and coefficient of variation (CV), multimodality and overlap between the size distribution modes were analyzed, according to:

I. Best K (BIC): The optimal number of Gaussian components (K) in the particle size distribution was determined using a Gaussian Mixture Model (GMM). The Bayesian Information Criterion (BIC) was used to select the model with the best trade-off between goodness-of-fit and model complexity.

II. Ashman's D: For testing the case of two Gaussian components ($K = 2$), the Ashman's D parameter was calculated as:

$$D = \frac{|\mu_1 - \mu_2|}{\sqrt{0.5\left(\sigma_1^2 + \sigma_2^2\right)}}$$

where μ_1 and μ_2 are the means and σ_1 and σ_2 are the standard deviations of the two modes.

Then,

- $D \geq 2 \rightarrow$ well-separated modes (clear bimodality)

- $1 \leq D < 2$ → partial overlap (weak bimodality)
- $D < 1$ → essentially unimodal

III. Overlap Coefficient (O): The overlap coefficient quantifies the shared area between two normalized distributions, given by

$$O = \int min[P_1(x), P_2(x)]dx$$

where P_1 and P_2 represent the probability density functions of the two Gaussian components. Values close to 0 indicate well-separated modes, while values close to 1 correspond to almost complete overlap.

Thus, in general, it follows that:

- Best $K > 1$, $D \geq 2$ and $O \leq 0.2$ → light bimodality
- Best $K > 1$, $1 \leq D < 2$ and $O \sim 0.3 - 0.6$ → weak/overlapping bimodality
- Best $K = 1$ or $D < 1$ → unimodality

To determine the values for Table S01, the following protocol was followed: GMM was applied to particle diameters values derived from SEM, obtained using ImageJ software. For each set of diameter values, models with K = 1–6 Gaussian components were fitted. For each K, the optimization was repeated using multiple initializations, and the solution with the highest logarithmic likelihood was retained. Model selection was based on the BIC, and the Best K corresponds to the model with the lowest BIC among the tested K values. The complete datasets

were used in each fit, with N = 447 (M1), 494 (M2), 318 (M3), 344 (C1), 375 (C2), and 396 (C3) particle diameters.

Table S01- Statistical data on SiO_2 particle sizes.

Sample	Mean ± SD (nm)	IQR	CV (%)	Best K (BIC)	Ashman's D	Overlap	Modal profile
C1	141 ± 35	40.2	25.5	2	1.07	0.49	Weak/overlapping bimodality
C2	320 ± 206	127.2	64.6	2	2.3	0.18	Clear bimodality
C3	418 ± 193	274.7	46.1	1	0.9	0.74	Unimodal
M1	94 ± 49	51.1	52.2	3	1.9	0.57	Partially overlapping
M2	100 ± 39	41.2	36.3	1	1.1	0.63	Unimodal
M3	134 ± 36	45.3	27.4	1–2	1.3	0.59	Weak shoulder
MA	245 ± 40	51.92	16.12	1–2	1.6	0.01	Weak shoulder

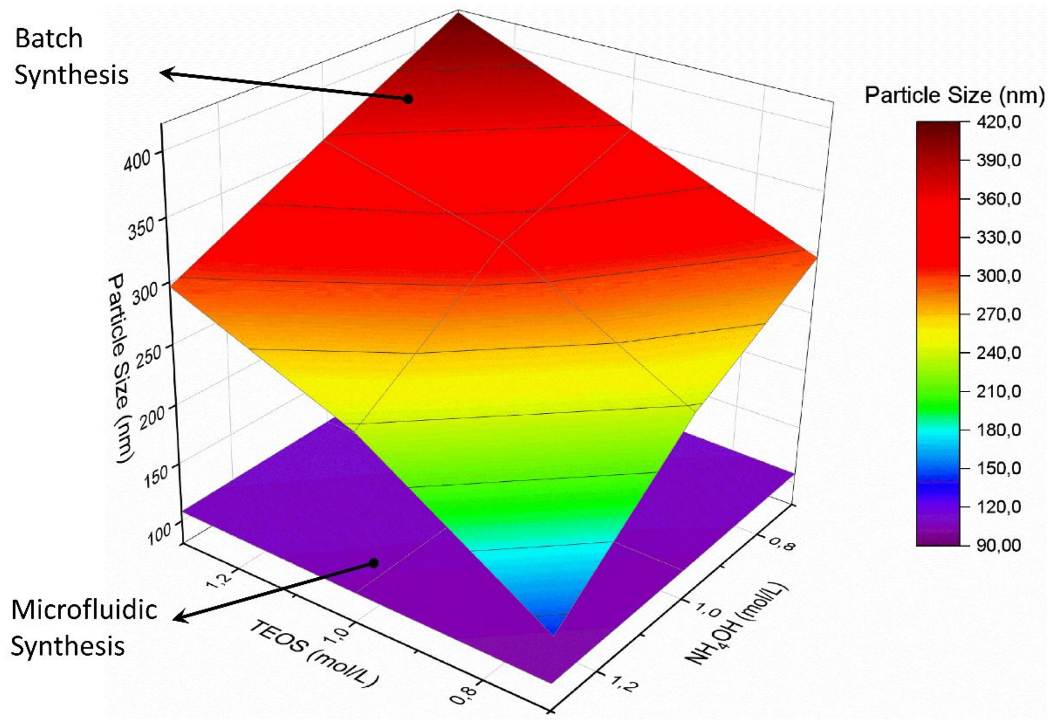

Figure S5- Three-dimensional surface plot with color map comparing particle size trends of batch and microfluidic syntheses as a function of TEOS and NH_4OH concentration. The particle size values are based on the statistical analysis reported in Table S01. The same color scale is used for both surfaces to allow quantitative comparison.

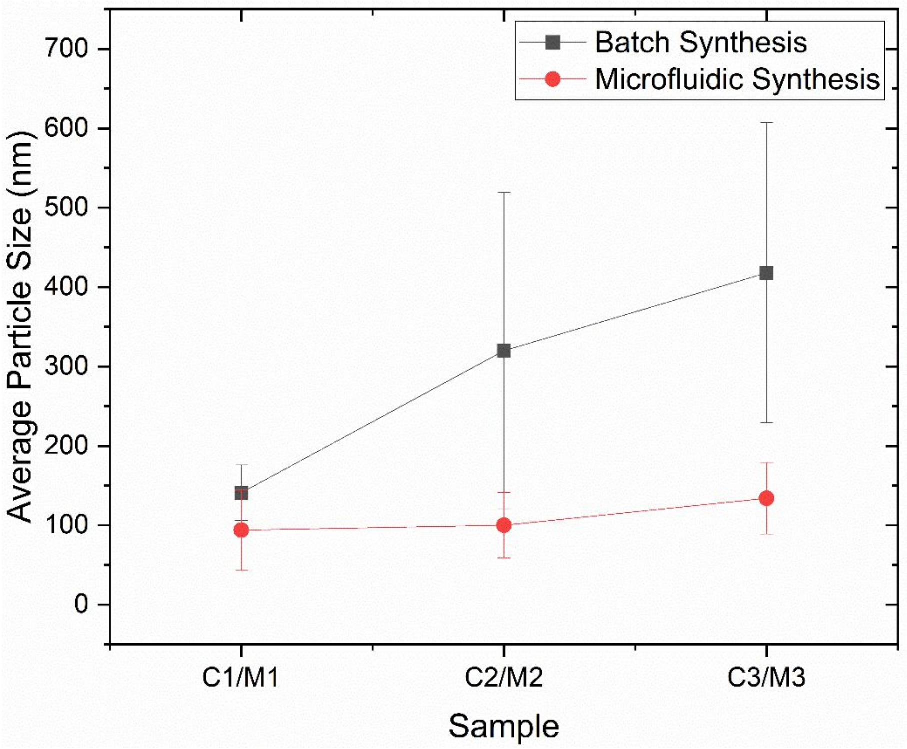

Figure S6- Average particle diameter for each sample. Symbols show the mean size determined from SEM measurements, and error bars represent the standard error of the mean. For samples with bimodal or multimodal size distributions, the mean is reported only as a compact descriptor and does not represent the modal diameters.
